# Morphological Characters and Transcriptome Profiles Associated with Black Skin and Red Skin in Crimson Snapper (*Lutjanus erythropterus*)

**DOI:** 10.3390/ijms161126005

**Published:** 2015-11-12

**Authors:** Yan-Ping Zhang, Zhong-Duo Wang, Yu-Song Guo, Li Liu, Juan Yu, Shun Zhang, Shao-Jun Liu, Chu-Wu Liu

**Affiliations:** 1 College of Life Science, Hunan Normal University, Changsha 410081, China; zhangyanping6000@163.com; 2 Fisheries College, Guangdong Ocean University, Zhanjiang 524088, China; aduofa@gmail.com (Z.-D.W.); gysrabbit@163.com (Y.-S.G.); zjouliuli@163.com (L.L.); yujuan0123@foxmail.com (J.Y.); apple896@126.com (S.Z.)

**Keywords:** *Lutjanus erythropterus*, skin color difference, transcriptome, gene expression

## Abstract

In this study, morphology observation and illumina sequencing were performed on two different coloration skins of crimson snapper (*Lutjanus erythropterus*), the black zone and the red zone. Three types of chromatophores, melanophores, iridophores and xanthophores, were organized in the skins. The main differences between the two colorations were in the amount and distribution of the three chromatophores. After comparing the two transcriptomes, 9200 unigenes with significantly different expressions (ratio change ≥ 2 and *q*-value ≤ 0.05) were found, of which 5972 were up-regulated in black skin and 3228 were up-regulated in red skin. Through the function annotation, Gene Ontology (GO) and Kyoto Encyclopedia of Genes and Genomes (KEGG) pathway analysis of the differentially transcribed genes, we excavated a number of uncharacterized candidate pigment genes as well as found the conserved genes affecting pigmentation in crimson snapper. The patterns of expression of 14 pigment genes were confirmed by the Quantitative real-time PCR analysis between the two color skins. Overall, this study shows a global survey of the morphological characters and transcriptome analysis of the different coloration skins in crimson snapper, and provides valuable cellular and genetic information to uncover the mechanism of the formation of pigment patterns in snappers.

## 1. Introduction

As one of the most diverse phenotypic characteristics in vertebrates, coloration plays numerous adaptive functions like camouflage, predator deterrence and species recognition [1,2]. Skin coloration can be influenced by many factors, such as genetics, diet, environmental or healthy condition, *etc.* [3]. Nevertheless, genetic is still the major determination, the kind of pigmentation related genes and their variant expressions are the major reason of diverse form coloration [1]. In mammalian systems, melanophores are the only chromatophore type found in their skin. In contrast, several kinds of chromatophores are found take part in the formation of variety coloration in teleost, including melanophores (melanin granules), xanthophores (pteridine or carotenoid granules), iridophores (guanine), leucophore (unknown) and erythrophores (carotenoids and pteridine) [4,5,6,7,8,9]. As most of the pigment related genes were first identified in laboratory mice (genus *Mus.*), to date, most of the known pigmentation genes are genes responsible for producing melanin [10,11,12,13,14,15], even in the teleosts system. Only a few studies about genetic of xanthophore [16] and iridophore [17,18] have been reported recently. However, pigmentation is an important economic trait for fish, achieving a uniform and bright coloration is crucial for fish farms.

With the advantage of low cost and speed, massively parallel sequencing (Illumina) RNA-Seq analysis is now the most convenient method to find out new genes and investigate gene expression patterns of non-model organisms, especially for species of which the whole genome sequence is not yet available, such as sheep [19], spider [20,21], *Ischnura elegans* [22], Yesso Scallop [23], *etc.* To date, several studies have reported on the gene expression profile of different coloration patterns of fresh-water fish like common carp [3,24], cichlids [25] and zebrafish [17]. These studies have found that signaling pathway such as Wnt (wingless-type MMTV in integration site family), MAPK (mitogen-activated protein kinase) and cAMP (cyclic adenosine monophosphate) were conserved melanin-synthesis related pathways in vertebrates. Higdon *et al.* [17] have proposed the purine synthesis and phosphoribosyl pyrophosphate might take part in the guanine production in zebrafish, the latter is a basic component of iridophore. However, studies about the genetic profiles on the skin of seawater fish species remain scarce. Considering from the morphology perspective, body coloration differences were mainly caused by the type, density and distribution of chromatophores [5,8,9]. From the cellular level, which chromatophores and how they involved in the formation of variety colorations, and from the genetic level, which genes correlated with the different pigmentations is still poorly understood.

In the South China Sea (SCS), there are about 20 indigenous species of genus *Lutjanus* present, which are economically important and a significant source of food for developing countries around SCS [26,27]. All of them have diagnostic color patterns that are primary taxonomic identification characters. To date, most studies about *Lutjanus* were mainly focused on their phylogenetic relationships [28,29]. Interestingly, Wang *et al.* [27] have found that as a kind of coral reef fish, there might be some relevance between the coloration and speciation in *Lutjanus*. However, there is little knowledge about the formation of these diverse pigment patterns in *Lutjanus*. Crimson snapper (*Lutjanus erythropterus*), which is distributed over the Indo-West Pacific and habitats throughout coral reef and hard-bottom, is one of the most economically important fish of SCS [30]. A suitable model for exploring the genetic basis of skin coloration is provided by the distinct skin colors of crimson snapper. The morphological characters of crimson snapper are very conservative and simple—the whole body is light red with more intense pigment on the back and a big black dot on the caudal trunk. To better understand the cells and genetic factors that influence the pigmentation formation, in this study, we utilized Stereomicroscope and Transmission Electron Microscopy (TEM) technology to observe the chromatophores morphology of black skin and red skin in crimson snapper. RNA-Seq was conducted on the two color skins of crimson snapper to compare their gene expression profiles. The purpose of this study is to provide basic information about the color difference from the cellular level, and identify the genes potentially related to the color determination of crimson snapper as well as find out the genetic differences between the two different color traits. Understanding this will not only enrich the information of skin color varieties in fish but also help the selection of fish species with consumer-favored coloration from the genetic level. On the other hand, our ultimate aim is to provide some candidate pigmentation genes to investigate the correlation between coloration and sympatric speciation in *Lutjanus* fishes.

## 2. Results

### 2.1. Chromatophore Distribution of Black Skin and Red Skin

**Figure 1:**
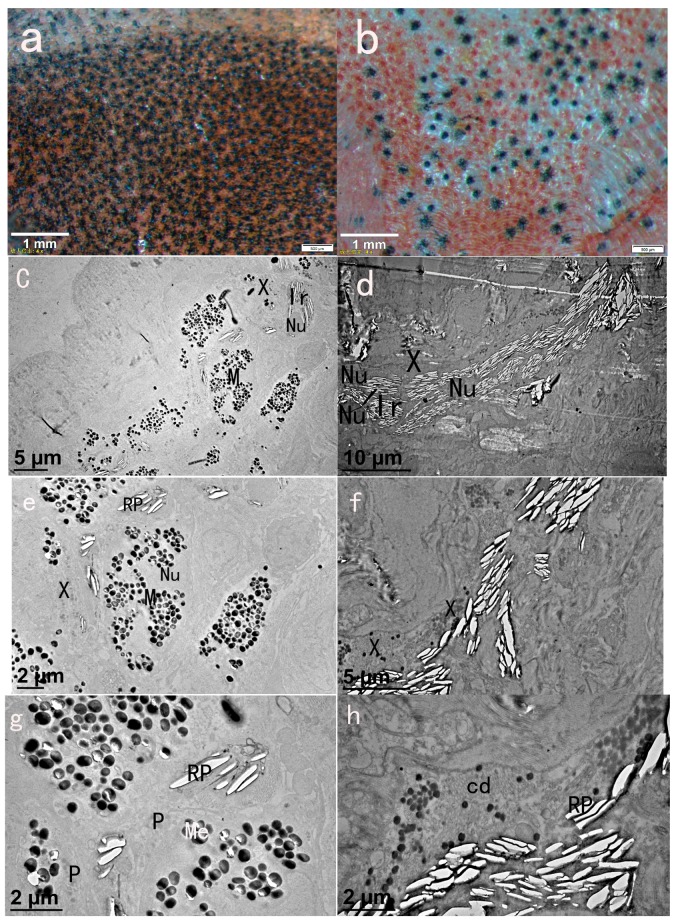
Skins of crimson snapper under Stereomicroscope and Transmission Electron Microscopy (TEM): (**a**) vlack skin (stereomicroscope); (**b**) red skin (stereomicroscope); (**c**) black skin, M: melanophore, Ir: iridophore, Nu: nucleus (TEM); (**d**) red skin, M: melanophore, Ir: iridophore, X: xanthophore, Nu: nucleus (TEM); (**e**,**g**) black skin, M: melanophore, Ir: iridophore, X: xanthophore, Me: melanosome, P: pterinosome, Nu: nucleus (TEM); and (**f**,**h**) red skin, M: melanophore, Ir: iridophore, X: xanthophore, Nu: nucleus, cd: carotenoid droplet, Me: melanosome, P: pterinosome, RP, reflecting plate (TEM).

From the Stereomicroscope observation of black skin and red skin crimson snapper, we found that there are three types of chromatophores: melanophore, iridophore and xanthophore in the fish skin, as shown in Figure 1. The main difference between the two colorations was in the type and quantity of the pigment cells, the black skin was mainly distributed by melanophore, while the red skin was based on xanthophore and iridophore.

Because of their black color and stellated shape, melanophores were the most easily observed cell type. Under TEM, melanophores were about 10 μM long, 4–6 μM in diameter, oval or dendritic shaped, with numerous melanin-bearing granules, called melanosome, filled in the cytoplasm. The melanosome varied from round to ellipsoidal and measured about 0.5 μM in diameter. Iridophores contributed to white- or silver-color region, they were difficult to detect when observing whole skin dissections. Under TEM, Iridophores were easily observed, they were dermal reflective cells, elliptical or shuttle shaped, near other pigment cells. Numbers of thin flat and reflective platelet filled in the iridophores. Under Stereomicroscope, xanthophores have a similar stellated shape to melanophores, which were different from the round shape of erythrophores [31]. At the same time, they displayed yellow to orange color, which was caused by the type and amount of pigment they contained. From the TEM results, pterinosome and carotenoid droplet were present in the cytoplasm of xanthophore. Carotenoid droplets were present widely in xanthophores in the red skin (Figure 1h). They were about 0.1 μM in diameter, oval vesicle and contained carotenoid pigment. Pterinosomes were bigger spherical vesicles contained densely stained contents. This kind of xanthophore was mainly distributed in the black skin (Figure 1e).

### 2.2. Sequencing and Assembly of the Black Color Skin and Red Color Skin Transcriptomes

Sequencing generated 52,873,586 raw reads from red fish skin and 54,232,958 raw reads from black fish skin, after removing repetitive, low-quality, and low-complexity reads, 49,531,098 clean reads with 50.73% GC percentage and 51,438,110 clean reads with 49.84% GC percentage were obtained from red color skin and black color skin, respectively. Then, after assembling these clean reads into unigenes, 122,508 and 142,792 unigenes with mean length of 613 and 622 bp were yielded from red color skin and black color skin, respectively (as shown in Table 1). Finally, 6803 and 7914 unigenes with sequence length greater than 2000 nucleotides were obtained from red color skin and black color skin, respectively. These unigenes were annotated with National Center for Biotechnology Information non-redundant protein database (NR), UniProt/Swiss-Prot, Cluster of Orthologous Groups of Proteins (COG), Gene Ontology (GO) and the Kyoto Encyclopedia of Genes and Genomes (KEGG) databases and 49,935 genes, 44,122 genes, 36,976 genes, 15,174 genes and 33,038 genes were obtained, respectively.

**Table 1:** Summary of transcriptome sequencing and assembly for black skin and red skin of crimson Snapper.

Parameters	Back Skin	Red Skin
Total raw reads	54,232,958	52,873,586
Total clean reads	51,438,110	49,531,098
Total clean nucleotides (bp)	4,629,429,900	4,457,798,820
Q20 percentage	94.41%	94.35%
N percentage	0.00%	0.00%
GC percentage	49.85%	50.73%
Total length	104,322,550	89,828,794
N50 (bp)	881	888
Unigenes	142,792	122,508
Mean length (bp)	622	613

### 2.3. Genes Highly Expressed in Fish Skins

The RPKM (reads per kilobase of xon model per million mapped reads) value of each gene was computed to represent its expression level in different tissues. Top expressed genes in each tissue were identified before Differentially Transcribed Genes (DTGs) were found between them. The top 10 genes (shown in Table 2) that were highly expressed in black and red colored fish skins were analyzed. From the results, we found that genes which encoding for ribosomal proteins were accounted for the majority of the top 10 highly expressed genes in both color skins, ⁶⁄₁₀ and ³⁄₁₀ in black skin and red skin, respectively. This result is in accordance with Higdon [17], whose study found that the top expressed genes in pigment cells of zebrafish were also genes encoding for ribosomal proteins. Different from the highest expression gene was the ribosomal protein genes in the black skin, the top six highly expressed genes in the red fish skin were creatine kinase M-type, fructose-bisphosphate aldolase, myosin light chain 3, cytochrome c oxidase subunit 1, NADH dehydrogenase subunit 5 and parvalbumin.

**Table 2:** Top 10 highly expressed genes in fish skin.

Back-Skin RPKM *	Gene Name	Red-Skin RPKM	Gene Name
16,496.2127	40S ribosomal protein S25-like	13,630.6212	Creatine kinase M-type
12,175.4483	hypothetical protein LOC100078788	13,504.9082	Fructose-bisphosphate aldolase
10,046.855	ribosomal protein L27a	12,425.8508	myosin light chain 3
10,041.9354	40S ribosomal protein S15	9826.1597	cytochrome c oxidase subunit 1
9797.9735	uncharacterized protein LOC100849392	8020.0354	NADH dehydrogenase subunit 5
8880.9287	putative ribosomal protein L14	6576.8346	parvalbumin
8046.257	ribosomal protein L18	9305.5688	40S ribosomal protein S25-like
914.1748	unnamed protein product	6265.6913	60S ribosomal protein L41
7404.5547	60S ribosomal protein L8	5994.7734	ribosomal protein L27a
7289.4817	unnamed protein product	5751.3138	myosin light chain 2 polypeptide

* RPKM: reads per kilobase of exon model per million mapped reads, which was used to represent the gene’s expression level in one tissue.

### 2.4. Recognition of Differentially Transcribed Genes (DTGs) in Black Skin versus Red Skin

**Figure 2:**
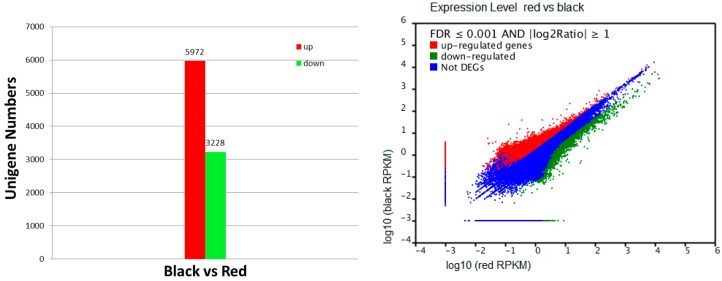
Differentially expressed genes in the two skins. The number of differentially transcribed genes (DTGs) identified in each library contrast applying a threshold of the ratio change and a *q*-value of <0.05. The left red/green column represents genes up-/down-regulated in different color skins. The right panel shows scatter plot of DTGs (false discovery rate (FDR) ≤ 0.001 and log2 Ration ≥ 1) illustrating the full set of genes sampled. Red points: up-regulated genes; Green points: down-regulated genes; Blue points: not DTGs.

In total, 117,249 differentially expressed transcripts between the two skins were found, and the number was reduced to 9200 after choosing the *p* < 0.0001 with the absolute value of log2 abundance ratio of ≥1 and an false discovery rate (FDR) of ≤0.001 as the cutoff (as shown in Figure 2). After comparing these genes with the NR, Swiss-prot, COG database, GO, and KEGG database, a total of 4350 genes were annotated, of which 2325 were up-regulated and 2025 were down-regulated in black skin in contrast with red skin. However, there are still a large number of differentially transcribed genes that could not be annotated, including some genes with high expression. In total, 4850 differentially expressed genes were considered as novel genes, among them 3647 were up-regulated and 1203 were down-regulated in skin of black color compared with skin of red color.

### 2.5. Functional Enrichment of Differentially Transcribed Genes (DTGs)

**Figure 3:**
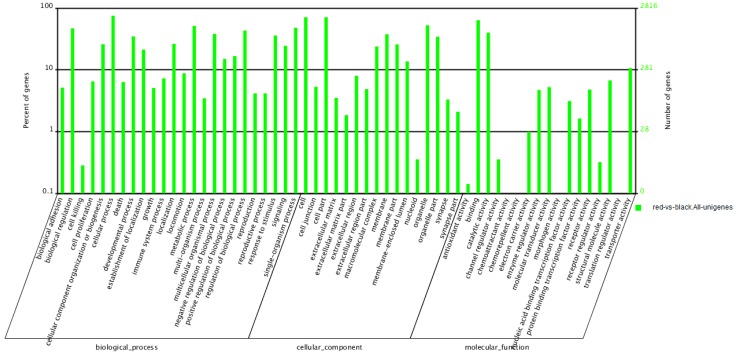
Gene Ontology (GO) functional classification of Differentially Transcribed Genes (DTGs) in red skin *versus* black skin.

After being GO annotated, the DTGs were classified into 57 GO terms, including 24 biological processes, 16 cellular components, and 17 molecular functions (as shown in Figure 3). Cellular process and metabolic process are the two largest categories in biological process. The largest two categories in molecular functions are binding and catalytic activity. For the cellular components, the most abundant categories are cell and cell part. However, the GO term of pigmentation do not appear to be significantly enriched in the differentially transcribed genes.

Of the 4350 known DTGs in black *versus* red skin, 3455 were annotated in the KEGG database. These DTGs were participated in 249 pathways, of which 26 (Table 3) were significantly enriched (*q*-value < 0.05). Among the 26 pathways, almost all the DTGs involved in Oxidative phosphorylation (75), Proteasome (28), Glycolysis/Gluconegenesis (34), Citrate cycle (24) and Cardiac muscle contraction (156) pathways are down-regulated in the black skin. However, at the same time, most of DTGs involved in tyrosine metabolism (18) were up-regulated in the black skin.

**Table 3:** Kyoto Encyclopedia of Genes and Genomes (KEGG) functional analysis of DTGs in black skin *versus* red skin.

Pathway	DTGs * with Pathway	*q*-Value	Pathway ID
Dilated cardiomythy	301	1.04 × 10^−65^	ko05414
Hypertrophic cardiomyopathy(HCM)	291	8.36 × 10^−63^	ko05410
Cardiac muscle contraction	156	2.02 × 10^−24^	ko04260
Alzheimer’s disease	150	2.22 × 10^−20^	ko05010
Oxidative phosphorylation	75	2.85 × 10^−18^	ko00190
Parkinson’s disease	80	1.72× 10^−17^	ko05012
Calcium signaling pathway	150	3.53 × 10^−11^	ko04020
Proteasome	28	5.07 × 10^−9^	ko03050
Huntington’s disease	115	1.21 × 10^−7^	ko05016
Viral myocarditis	118	3.68 × 10^−7^	ko05416
Vascular smooth muscle contraction	149	4.29 × 10^−6^	ko04270
Focal adhesion	202	1.32 × 10^−5^	ko04510
Amoebiasis	153	1.04 × 10^−4^	ko05146
Arrhythmogenic right ventricular cardiomyopathy	75	2.47 × 10^−4^	ko05412
Metabolic pathways	400	2.70 × 10^−4^	ko01100
Regulation of actin cytoskeleton	220	3.05 × 10^−4^	ko04810
Protein digestion and absorption	79	3.87 × 10^−4^	ko04974
Tight junction	153	8.05 × 10^−4^	ko04530
Glycolysis/Gluconeogenesis	34	8.66 × 10^−4^	ko00010
Salmonella infection	120	1.66 × 10^−3^	ko05132
Phenylalanine, tyrosine and tryptophan biosynthesis	8	1.66 × 10^−3^	ko00400
Arginine and proline metablism	30	4.50 × 10^−3^	ko00330
Citrate cycle (TCA cycle)	24	4.80 × 10^−3^	ko00020
Phenylalanine metabolism	13	5.16 × 10^−3^	ko00360
Alanine, aspartate and glutamate metabolism	21	9.60 × 10^−3^	ko00250
Tyrosine metabolism	18	2.88 × 10^−2^	ko00350

* DTGs, differetially transcribed genes, which was identified by the DEGseq package. DTGs between the two samples were selected with the following filter criteria: log2 transcript abundance ratio ≥1 and FDR (false discovery ratio) ≤0.001.

### 2.6. Differential Expression of Known Pigmentation Genes

According to the ensemble database [32], 97 genes are participated in the pigmentation category in the zebrafish. After a BLAST search with the 97 pigmentation genes, a total of 84 of aforementioned genes (with *E* value < 1 × 10^−5^) were detected in crimson snapper skin in this study (data seen in Table S1). Considering the RPKM of these genes, we found 16 genes showed significant highly expressed in black skin and three genes with significantly high expression in red skin. Among the significant differentially expressed genes, sodium calcium transporter 45a2 (*SLC45a2*) was found to be the most highly expressed gene in black skin, followed by oculocutaneous albinism II (*OCA2*), sodium calcium transporter 24a5 (*SLC24a5*), pre-melanosomal protein a (*PMELa*) and HMG box transcription factor Sox9b (*sox9b*). The three genes significantly high expression in red skin were choroideremi (*chm*), ADP-ribosylation factor-like (*arl6*) and aldolase a, fructose-bisphosphate, a (*aldoaa*).

### 2.7. Confirmation of RNA-Seq Identified DTGs by Quantiative Real-Time PCR (qRT-PCR)

**Figure 4:**
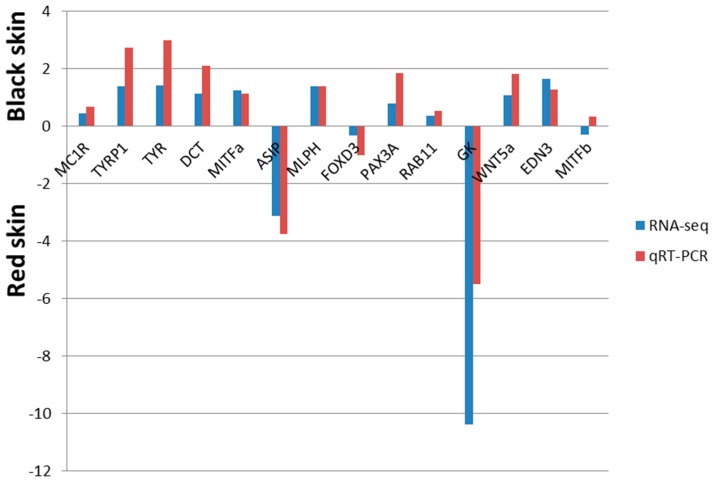
Comparison of gene expression patterns obtained using comparative transcriptome analysis and qRT-PCR (Quantiative real-time PCR). RNA-seq fold changes are computered directly for each sample comparison (*i.e.*, log2(black RPKM/red RPKM)) qRT-PCR fold changes are calculated by first normalizing expression relative to *β-actin*, followed by the log transformation. Gene abbreviations: melanocortin 1 receptor (*MC1R*); tyrosinase-related protein 1 (*TYRP1*); tyrosinase (*TYR*); dopachrome tautomerase (*DCT*); microphthalmia-associated transcription factor a (*MITFa*); agouti signaling protein (*ASIP*); melanophilin (*MLPH*); forkhead box D3 (*FOXD3*); paired box protein 3a (*PAX3a*); member RAS oncogene family (*RAB11*); glycerol kinase (*GK*), wingless-type MMTV integration site family member 5A (*WNT5a*); endothelin 3 (*EDN3*); microphthalmia-associated transcription factor b (*MITFb*).

In order to test the reliability of RNA-Seq datas, 14 pigment genes were randomly selected for qRT-PCR to check their expression in the two skins. After comparing the gene expression patterns in the qRT-PCR with the data of RNA-Seq (shown in Figure 4), results showed that expression patterns of the selected genes obtained from the qRT-PCR was nearly in accordance with the RNA-Seq data, except the *MITFb* gene. Combined with the results of the two methods, we found that melanin-related genes showed higher expression in black skin and guanine-related genes showed higher expression in red skin, both of them certified the different expression patterns of pigment-related genes in diverse color skins.

## 3. Discussion

Animal coloration plays important roles in communication, ecological interactions and even speciation [16]. Studies have found that diverse body colorations are mainly controlled by the development and patterning of pigment cells, of which genetic was the major determination. Thus, in this study, firstly, we observed the morphology of black skin and red skin of crimson snapper. Secondly, by Illumina sequencing technology, we studied the genetic profiles of black skin and red skin of crimson snapper from transcriptome level, and by contrast, 9200 significantly DTGs were found. The identified DTGs between the two color skins will not only help us to understand the molecular mechanism of skin color differences, but also provide us valuable gene information for exploring the relevance of speciation and pigmentation in this species. Compared to the recent RNA-Seq research on common carp skin [33], more DTGs were found in our study. The first reason might be less use of analysis technology, as the aim of this study is to identify a large number of candidate genes for subsequent analysis, only one conventional technology DEGseq [34] was used to identify the DTGs. The second reason might be the diverse chromatophores composition of crimson snapper skins, as shown in Figure 1.

In this study, we found ribosome protein genes were accounted for the highly expressed genes in each tissue, which suggested ribosome proteins might play an important role in the fish skin formation. Previous study has reported that in the transcriptome of zebrafish pigment cells, four of the top five most highly expressed genes were ribosomal proteins [17]. A similar finding was also reported on the transcriptome analysis of sheepskin [19]. Studies have proved that highly expressed levels of ribosome proteins related genes not only revealed the high rates of protein translation in organism [35], but also have some correlation with the mouse black coat color [36]. Combined with the more highly expressed of ribosome protein genes in black skin, so we inferred the ribosomal proteins genes might involve in the formation of the black skin coloration of crimson snapper. However, further studies are needed to deliberate its exact function. While, different from the black skin, in the red skin, creatine kinase M-type, fructose-bisphosphate aldolase (*FBA*), cytochrome c oxidase subunit 1 (*COX1*), Glyceraldehyde-3-phosphate dehydrogenase (*GAPDH*), NADH dehydrogenase subunit 5 (*NADH5*) and parvalbumin were also showed very high expression, all of these genes were found to be iridophore-related genes in zebrafish [17]. Meanwhile, Fan *et al.* [19] has found that the expression of *NADH5* and *COX1* were very high in the white skin of sheep, as both of them were genes encoding for the enzymes responsible for oxidative and dehydrolytic, so they presumed that the high expression of them might imply strong metabolism characteristic of iridophores. However, the function of the *NADH5* and *COX1* in crimson snapper needs further elucidation. As an activator to tyrosine kinase [37,38], fructose-bisphosphate aldolase (*FBA*) and Glyceraldehyde-3-phosphate dehydrogenase (*GAPDH*) were constituents of the glycolytic/gluconeogenesis pathway. However, from the recent study [17], we known that *GAPDH* might play important roles from the guanine synthesis to the formation of iridophore pigment contained organelles. All of these results indicated these genes might play some role in the red skin in the energy metabolism or synthesis of guanine, further work is still needed to determine how these genes function.

After the GO and KEGG analyses of DTGs, the most clustered groups of DTGs were consistent with previous works about fish, like zebrafish [17], Midas cichlids [25] and common carp [3,24,33]. After the GO analysis of the DTGs, we found that most of the down-regulated genes were enriched in pathways like Glycolysis/Gluconeogenesis, Citrate cycle, Oxidative phosphorylation, Cardiac muscle contraction and Proteasome, which implied the participation of these pathways in the formation of red skin. In zebrafish, several TEM studies about fish skin have showed the existences of stacks of guanine plates in iridophores [5,9,39], and glycolysis and citrate cycle pathway were found to be key participators with the extensive guanine synthesis [17]. Combined with our TEM results (Figure 1d), a number of reflecting plates were found in the iridophores; thus, higher expression of genes with these pathways might be in accordance with the higher requirement of guanine for the reflective iridophore pigment in red fish skin crimson snapper. Further work is still needed to determine how these pathways coordinate the up-regulation of guanine synthesis in iridophores.

To test the reliability of the RNA-Seq datas, 14 genes were chosen randomly for qRT-PCR, including *WNT5a*, *MC1R*, the tyrosine gene family (*DCT*, *TYR* and *TYRP1*), *etc.* As shown in Figure 4, the expression pattern of the pigment-specific genes from qRT-PCR was almost in accordance with the RNA-Seq result except the *MITFb* gene, and the expression level of the two methods was nearly in accordance, with only slight differences, indicting the reliability of the transcriptome data. Several studies [24,40,41] have reported that WNT signaling pathway taken part in the synthesis of melanogenesis in teleost as well as mammals. *WNT5a*, a non-canonical Wnt protein family gene which was found located in the matrix and precortex cells in the hair follicles of mice [42,43], was 1.1-fold up-regulated in the black skin. Interestingly, in the recently RNA-Seq study about the common carp skin, the member of Wnt protein family showed higher expression in YRC was *WNT5b*. Brassch *et al.* [44] have found that due to the fish-specific genome duplication (FSGD), 75% of the melanogenic enzymes were found to be duplicated, and three different fated for duplicated genes were observed in [45]. So further studies should be conducted to determine whether species-specific evolution variation exist in *WNT5* duplicates in crimson snapper. *MITF* [46,47], the master regulator of melanophore identity, was 1.2-fold up-regulated in the black skin, and the downstream gene of *MITF*, tyrosinase gene family [44], which is well known to take part in the enzymatic conversion of tyrosine to melanin, were also showed up-regulation in the black skin. However, agouti signaling protein (*ASIP*) [48], the gene blocked the melanogenesis by antagonizing the binding of α-*MSH* to *MC1R*, showed a higher expression in red skin in either method. All of these results not only proved the credibility of the transcriptome data but also indicted the conservation of these pigmentation genes in teleost.

According to the comparison between known zebrafish pigmentation genes with the transcriptome data of this study, majority of the pigmentation genes could be found in the snapper fish. In addition, we found most of the known pigmentation genes have shown significantly different expression patterns between the two tissues. Such as sodium calcium transporter 45a2 (*SLC45a2*) showed higher expression in black skin in contrast with red skin, with *OCA2*, sodium calcium transporter 24a5 (*SLC24a5*), pre-melanosomal protein a (*PMELa*) and sox9b were followed. Studies have found *SLC45a2* performed some role for organelle pH homestsis in pigment cells [49,50] and *in situ* analyses have also revealed its enriched expression in melanophores in zebrafish [49,50,51]. *OCA2*, together with the *SLC45a2*, *TYRP1*, *TYR* also belongs to the typical melanin synthesis pathway [52]. *PMELa*, encodes for a pigment cell-specific protein that might take part in the formation of fibrillar sheets contained in the melanosome [53]. In former RNA-Seq studies on cichlids [25] and stickleback [54], the expression of these genes was also found to be higher in dark bars. These results revealed the conservation of pigmentation genes across various species in term of sequences and functions.

From the cellular level, we found that the differences in the two colorations depending primarily upon the density and distribution of the chromatophores, in the black skin melanophores account for the major, and in the red skin leaving the iridophores and xanthophores the major. However, from the genetic level, after analyzing of the two skin transcriptomes, different expressed candidate pigmentation genes were mainly enriched in the pathways of melanin and guanine synthesis. There might be two reasons to explain this: Firstly, in crimson snapper, the xanthophores mainly filled with carotenoid droplets, which is a kind of pigment that vertebrates cannot synthesis endogenously [16]. Secondly, like Ng *et al.* [55] has found in *Nothobranchius* fish, xanthophores were functionally more related to the melanophores and most likely ontogentically closer to the melanocyte lineage than the iridophores.

In this study, approximately 50% of the DTGs did not find significant matches with known proteins after BLAST in public databases. The similar result has also occurred in the common carp skin transcriptome [3]. One of the possible elucidations might be that the non-model species possess many potential novel genes or transcripts that cannot be found in public databases. Therefore, further characterization of the unknown DTGs identified in the present study is required.

## 4. Experimental Section

### 4.1. Samples for Transmission Electron Microscopy (TEM) Observation and RNA Extraction

Samples of crimson snapper (*Lutjanus erythropterus*) (average weight 300 ± 10 g, average length 27 ± 1 cm) were obtained from a local fish market in ZhanJiang, Guangdong, China. Prior to experiments, the fish were kept in laboratory aquaria for 3 days for acclimatization with 14:10 h light and dark phase with temperatures between 26 ± 2 °C. All animal experiments were conducted according to the principles of the Laboratory Animal Management Ordinance of China. All fishes were anesthetized with MS-222 (Sigma, St. Louis, MO, USA) before being euthanized. Two different color skin samples were excised from the black dorsal site (B) and the red belly site (R) of the fish. Both the skin samples were cut into 0.5 mm × 0.5 mm × 1–2 mm and fixed in 2.5% glutaraldehyde and 2% paraformaldehyde in 0.1 M sodium cacodylate buffer, pH 7.4, 4 °C, 12 h. Then, they were post-fixed in 1% osmium tetrixude, in the same cacodylate buffer, followed by being gradually dehydrated with ethanol and embedded in Epon 812 (Sigma, St. Louis, MO, USA). Ultrathin sections were stained in uranyl acetate and lead citrate, and then observed with JEM-1400 Transmission electron microscope (JEOL, Tokyo, Japan) operating at 80 kV.

For the RNA extraction, fresh skin tissues of crimson snapper were collected and frozen in liquid nitrogen immediately and stored at −80 °C until further use. Different colored skin pieces were excised from red regions and dark regions as the previous described. The total RNAs were extracted with TRIzol^®^ Reagent (Invitrogen, Carlsbad, CA, USA). NanoDrop 2000 (Thermo Scientific, Wilmington, DE, USA) and gel electrophoresis were performed to assess the quantity and quality of total RNA. The total RNA used in the transcriptome analysis was the mixture of three samples. The mRNA were extracted using Sera-mag Magnetic Oligo (dT) (Thermo Scientific, Wilmington, DE, USA). After mixing with the fragmentation buffer, the mRNA was fragmented into short fragments, which were used as templates for the synthesized of the first strand of cDNA. After synthesis of the complementary strand, the doubled stranded cDNA were refined with the QianQuick PCR extraction kit (Qiagen, Dusseldorf, Germany) and connected with the sequence adapters. The fragments with lengths of ~200 bp were selected as templates. An Agilent 2100 Bioanaylzer (Agilent Technologies, Santa Clara, CA, USA) and ABI StepOnePlus Real-Time PCR System (ABI, Carlsbad, CA, USA) were used in quantification and qualification of the sample library.

### 4.2. Transcriptome Analysis

Transcriptome sequencing was carried out with Illiumina HiSeq 2000 RNA-Seq (Illumina, San Diego, CA, USA). The clean reads were acquired after removing reads with adapters, unknown nucleotides larger than 5% and the percentage of low quality bases (base quality ≤ 10) is more than 20%. Trinity [56] was used to conduct the *de novo* assembly of the transcriptome. Contigs, the longer fragments without N, were obtained by the combination of reads with overlapping length. Then, the different contigs were connected to get sequences that could not be extended on either ends, which were defined as unigenes. Some of the transcriptome data are shown in Table 1. The assembled sequences were compared against the NCBI non-redundant (Nr) protein database, Swiss-Prot, Kyoto Encyclopedia of Gene and Genomes (KEGG), and Clusters of Orthologous Groups (COG) of protein database using BlastX with an *E*-value of 1 × 10^−5^. The sequence direction of contigs was based on the best alignment results. A combination of BLAST, Blast2GO, KEGG and GO database was used for functional annotation. BLASTX alignment (*E*-value < 1 × 10^−5^) with the NT, NR, KEGG, Swiss-Prot, and COG database was conducted to get the associated gene name and gene ontology (GO) term accession number, and GO analysis was performed with the WEGO software [57].

The RPKM algorithm was used to quantify transcript abundance [58]. A log2 transcript abundance ratio ≥1 and an FDR (false discovery rate) ≤0.001 were used as the cutoff to define the significantly differences in transcript abundance. Based on the Hypergeometric distribution model, GO and KGEE ontology enrichment analyses were conducted on the differentially expressed genes. GO enrichment analysis was carried out for functional categorization of differentially expressed genes using GO-TermFinder software with an FDR adjust *p*-value of ≤0.05 as the limitation [59]. The biological complex behaviors of the differentially expressed genes were further analyzed according to the KEGG pathway database with *q* value ≤0.05 considered a significantly enriched pathway.

### 4.3. Quantitative Real-Time PCR Validate

qRT-PCR was performed to validate the transcriptome datas, 14 genes were selected randomly with *β-actin* used as the internal control. The first-strand cDNA was obtained from the previous total RNA using random primer and the Reverse Transcriptase M-MLV (Promega, Madison, WI, USA). Primers (shown in Table S2) were designed using the Beacon software. qRT-PCR was performed with SYBR^®^ Green PCR SuperMix (Thermo Scientific, Wilmington, DE, USA) and was done with the CFX 96 real-time PCR detection system (Bio-Rad, Hercules, CA, USA). The PCR was performed in a 10 μL reaction volume containing 0.5 μL of each primer (5 μM), 0.5 μL of cDNA, 5 μL of SYBR Green SuperMix, 3.5 μL ddH_2_O. The PCR cycle was 95 °C for 7 min, followed by 40 cycles of 95 °C for 10 s, 55 °C for 15 s, and 72 °C for 15 s. Each sample was run in three technical replicates and six biology replicates along with the internal control gene. Expression differences between black skin and red skin were assessed by first normalized to the expression level of β-actin, followed by the log transformation [17].

## 5. Conclusions

TEM and RNA-Seq were successfully employed to investigate the mechanism of body color variation in crimson snapper from the cellular and transcriptome levels. The chromatophore morphology and transcriptome information of crimson snapper skin is presented for the first time in this study. Three types of chromatophores were found in the skin, and from the cellular level, the color variation results from the amount of the three pigment cells distributed. The melanin synthesis and guanine synthesis related transcripts were abundantly detected in this study, and the up-regulation of pigment genes were in accordance with the amount of pigment cells distributed. We believe that the morphology and bio-information of transcriptome in this study will be of great value in understanding pigmentation formation by providing a large number of candidate pigmentation genes to uncover the mechanism of different colorations and will also be helpful in exploring the relevance between coloration and speciation of the *Lutjanus*.

## Supplementary Materials

Supplementary materials can be found at http://www.mdpi.com/1422-0067/16/11/26005/s1.
