# Supplementary figures and images for: Morphological Characters and Transcriptome Profiles Associated with Black Skin and Red Skin in Crimson Snapper (Lutjanus erythropterus)
Source: Int J Mol Sci. 2015 Nov 12;16(11):26991–7004. doi: 10.3390/ijms161126005 (PMC4661863; doi:10.3390/ijms161126005)

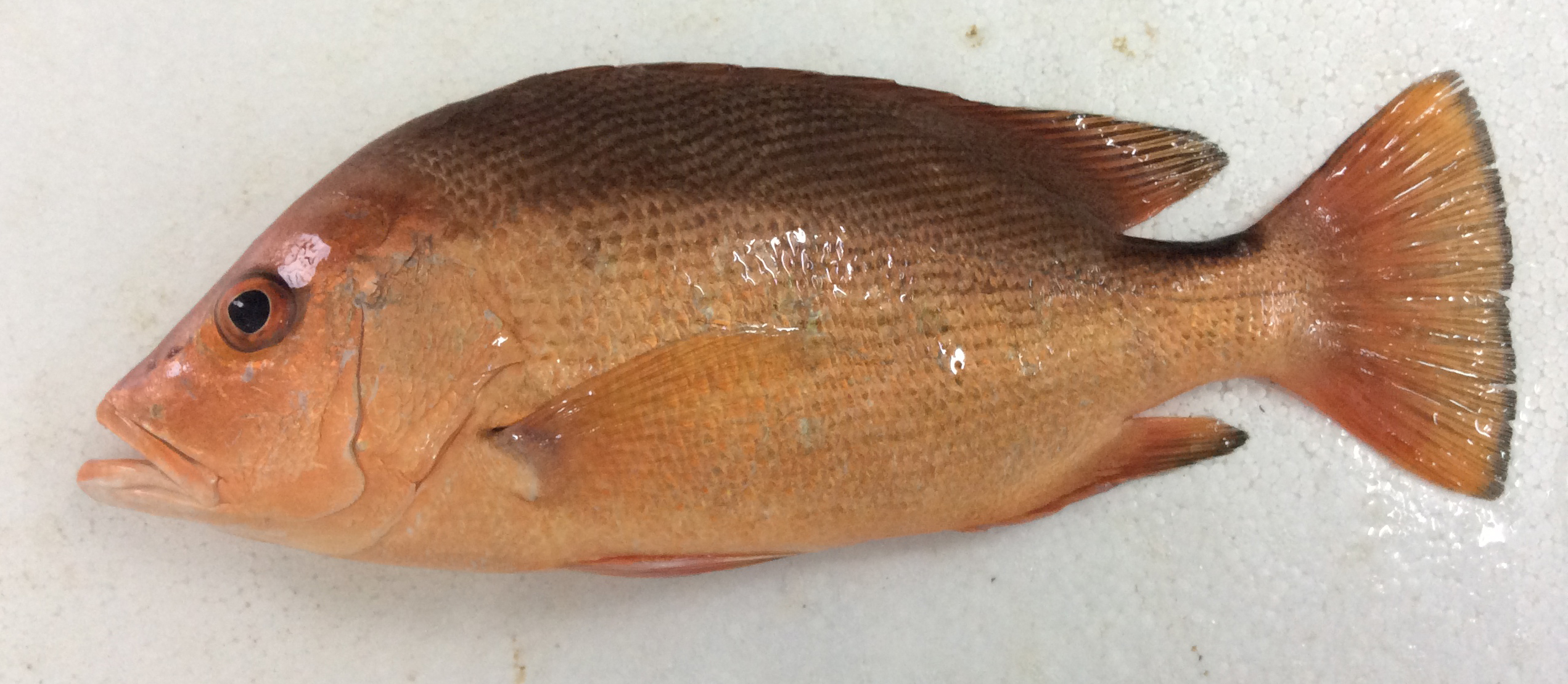

Supplement: Supplementary file 1 [file ijms-16-26005-s001.zip › ijms-102547-Supplementary Information/crimson snapper.jpg]
